# Vitamin C assisted synthesis of rGO–Ag/PANI nanocomposites for improved photocatalytic degradation of pharmaceutical wastes†

**DOI:** 10.1039/d1ra00171j

**Published:** 2021-03-24

**Authors:** S. Sravya, Dharmasoth RamaDevi, Neway Belachew, K. Eswara Rao, K. Basavaiah

**Affiliations:** Department of Inorganic and Analytical Chemistry, Andhra University Visakhapatnam-530003 India; AU College of Pharmaceutical Sciences, Andhra University Visakhapatnam-530003 India; Department of Chemistry, Debre Birhan University Debre Berhan Ethiopia neway.du@gmail.com neway@dbu.edu.et

## Abstract

A highly efficient visible light active polyaniline (PANI)/Ag composites grafted reduced graphene oxide (rGO–Ag/PANI) was prepared for the efficient photocatalytic degradation of paracetamol. The structural, morphological, and light absorption properties of the as-synthesized rGO–Ag/PANI were characterized by UV-Visible (UV-Vis) spectroscopy, Fourier transform infrared (FTIR) spectroscopy, powder X-ray diffraction (XRD), scanning electron microscopy (SEM), and transmission electron microscopy (TEM). Paracetamol was taken as a model water pollutant to investigate the photocatalytic degradation efficiency of the rGO–PANI/Ag nanocomposites under visible light radiation. The result shows the degradation of paracetamol to be 99.6% in the acidic medium (pH 5) and 75.76% in the basic medium (pH 9), respectively. The enhanced degradation efficiency is attributed to the synergetic effect of rGO, PANI, and Ag NPs in the nanocomposites. This synergy of the rGO–Ag/PANI is explained by the strong adsorption efficiency, charge separation, and light absorption in the visible region.

## Abstract 2

A highly efficient visible light active polyaniline (PANI)/Ag composites grafted reduced graphene oxide (rGO–Ag/PANI) was prepared for the efficient photocatalytic degradation of paracetamol.

## Graphical abstract

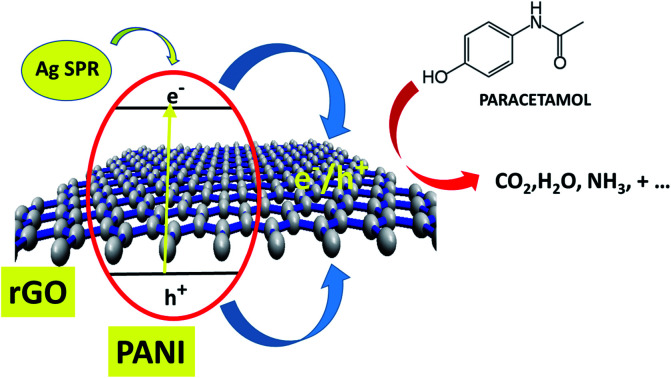

## 1 Introduction

During the last decade, water pollutants emerging from pharmaceutical, cosmetics, heavy metals, pesticides, industrial additives, and solvents are becoming new global water quality threats. Even trace level of a pharmaceutical drug in water has considerable health impact on the humans, animals, and aquatic species.^1,2^ The presence of pharmaceuticals in municipal wastewater, hospital wastes, and industrial effluents are the major sources of contaminants in drinking water.^3^ Particularly, paracetamol (chemically known as acetaminophen, 4-hydroxyacetanilide, 4-acetamidephenol or APAP) is one of the most common drugs used in the world as an analgesic and antipyretic drug.^4^ Paracetamol is highly accumulated in the aquatic environment and show adverse effects on the aquatic life and human health.^4^ Therefore, it is highly desirable to remove or reduce the paracetamol concentration below the recommended level before being discharged to the water bodies.

Many methods, such as chemical treatment, filtration, activated sludge, reverse osmosis, electrocoagulation, and advanced oxidation process, have been employed for the removal of paracetamol from contaminated water.^5–8^ Among the advanced oxidation (AO) processes, light-driven photocatalytic degradation has received considerable attention due to its cost-effectiveness, eco-friendly nature, and the absence of residual secondary pollutants.^9,10^ Therefore, the modification or synthesis of novel photocatalysts for this purpose is a prime concern in the field of AO. In this regard, multiple reports on the graphene-based multifunctional nanocomposites have been published for the photocatalytic degradation of water pollutants.^11,12^ Reduced graphene oxide (rGO) is a two-dimensional structure with strong suitability for electron delocalization and astonishing physicochemical properties prioritizing it for preparing nanocomposites.^13^ rGO supported metal/metal oxide nanoparticles have shown enhanced photocatalytic activity than their pristine form.^14^ Moreover, rGO blended with conducting polymers such as polyaniline (PANI) has been reported for the synergistic photocatalytic degradation of organic compounds.^15^

Polyaniline (PANI), among other conducting polymers, has shown promise for various technological applications because it adapts a facile preparation protocol, and shows interesting electrical conductivity, chemical stability, and catalytic properties.^16,17^ Furthermore, by doping or making composites with inorganic materials, the conductivity and catalytic properties of PANI can be enhanced. For example, Ma *et al.*^18^ have reported TiO_2_ modified rGO–PANI hybrid for the efficient photocatalytic removal of organic dye and production of hydrogen. This enhanced photocatalytic efficiency of the composite is ascribed to the extended spectral response in the visible region and separation of photogenerated charge carriers of TiO_2_ NPs. Similarly, Wu *et al.*^19^ prepared a tertiary composite of rGO–PANI–ZnO for improved photocatalytic degradation of methylene blue. The report demonstrates that a high surface area and charge separation efficiency of rGO and extended light absorption by PANI could be responsible for the enhanced photocatalytic efficiency of ZnO. Moreover, plasmonic metal nanoparticles, such as Ag, Au, Cu, Pt, and Ni, modified rGO or PANI were synthesized for improving photocatalytic activity.^20,21^ These metal nanoparticles because of their surface plasmon resonance have strong absorption efficiency in the visible region. Among these, Ag NPs due to biocompatibility, reasonable cost, stability, and easy reduction from salts using mild reducing agents have extensively been used to enhance the catalytic activity of the composite.^22^ Moreover, the irreversible agglomeration of Ag NPs is overcome by its immobilization on the surface of supporting materials.^12,23^ Herein, we are motivated to investigate the synergistic photocatalytic degradation efficiency of PANI–Ag NPs hybrids decorated rGO. To our knowledge, there is no report on utilizing rGO–Ag/PANI for the photocatalytic degradation of pharmaceutical waste, *i.e.* paracetamol.

Therefore, in the current study, tertiary nanocomposites of rGO, PANI, and Ag NPs were synthesized using a facile synthesis method. The partial reduction of graphene oxide (GO) to rGO and reduction of Ag^+^ to Ag^0^ were achieved by vitamin-C as a reducing agent. The detailed characterizations were carried out to explore the surface morphology, absorption, and structure of the synthesized composite materials. The improved photocatalytic degradation efficiency of rGO–Ag/PANI was weighed by the degradation of paracetamol. The effect of various experimental parameters such as solution pH, catalyst dose, and paracetamol concentration was carefully evaluated.

## 2 Experimental

### 2.1 Materials

Graphite flakes from Aldrich, potassium permanganate (KMnO_4_), hydrogen peroxide (H_2_O_2_), sodium nitrate (NaNO_3_), acetone, sodium hydroxide (NaOH), sulphuric acid (H_2_SO_4_), silver nitrate (AgNO_3_), aniline, ammonium persulphate (APS), hydrazine (N_2_H_4_), hydrochloric acid (HCl), ammonia solution (NH_3_, 25%) and ascorbic acid (vitamin-C) were purchased from Merck, India, and used for synthesis. Doubly distilled water was used throughout the whole synthesis process.

### 2.2 Synthesis of rGO–Ag/PANI nanocomposite

rGO–Ag/PANI nanocomposite was synthesized using vitamin C as a reducing agent. GO was prepared following the modified Hummers method (ESI.1†). For the synthesis of rGO–Ag/PANI nanocomposite, and 0.1 gram of GO was added to a 250 mL round bottom flask containing 100 mL deionized water (1 mg/1 mL) under ultra-sonication. Then, 5 mL of vitamin C (2 M) was added and the reaction was proceeded for 8 hours at 80 °C to get reduced GO (rGO) (Solution-1). In another reactor, 1 M HCl (20 mL) was added to 1 M of aniline (50 mL), and then 0.01 M APS (50 mL) was gently added. The reaction was allowed to proceed at 0 to 5 °C for 5 h before warming to room temperature to get the PANI (Solution-2).^23^ Fifty milliliters of AgNO_3_ (0.01 M) was added dropwise to the as-prepared PANI (Solution-2). Next, 5 mL of vitamin C (2 M) was added to the reaction mixture and the reaction progressed further for 30 min at constant stirring to get the PANI–Ag composite (Solution-3). Finally, PANI–Ag (Solution-3) was added dropwise to rGO (Solution-1) with a gentle stirring for 12 h at room temperature. The precipitate was collected by centrifugation, washed three times, and dried under vacuum at room temperature to obtain the as-prepared rGO–Ag/PANI.

### 2.3 Characterizations

The UV-Visible absorption spectra were recorded using a Shimadzu 2450 – SHIMADZU spectrometer. The sample was diluted with deionized water and it was introduced into a UV-Visible Shimadzu 2450 – SHIMADZU spectrometer for characterization of a sample. X-ray diffraction (XRD) patterns were recorded using a PANalytical X'pert pro diffractometer at 0.020 s^−1^ scan rate with Cu-kα radiation (1.5406 Å, 45 kV, 40 mA) and 2*θ* ranging from 10° to 90°. The powder samples were thoroughly mixed with KBr and pressed into thin pellets for measuring the Fourier transform-infrared (FTIR) spectra of the samples. The readings were recorded over the range of 400–4000 cm^−1^. Transmission electron microscopy images were obtained at an accelerating voltage of 200 kV (transmission electron microscope from JEOL, Japan). Scanning electron microscopy (Hitachi – scanning electron microscope S-3700N) images were acquired with an electron probe analyzer.

### 2.4 Photocatalytic degradation of paracetamol

The experiments on catalytic degradation efficiency of rGO–Ag/PANI nanocomposite was carried out using a paracetamol solution. Particularly, 50 mg of rGO–Ag/PANI nanocomposites was suspended in 50 mL of paracetamol solution (50 mg L^−1^). The sample initially was stirred in dark for 30 min and then exposed to visible light for catalytic degradation. Samples from the flask were withdrawn after the specified time of reaction and the residual undegraded paracetamol concentration was determined by a UV-Visible spectrophotometer at *λ*_max_ = 245 nm. The reaction was monitored by observing the change in absorbance peak of paracetamol at 245 nm in acidic, basic, and neutral medium with a different concentration ratio of paracetamol and rGO–Ag/PANI nanocomposites. The pH of the solution was adjusted by NaOH (0.01 M) for basic and HCl (0.01 M) for acidic medium. The percentage of paracetamol degradation (*D*%) was calculated by:*D*(%) = (*C*_0_ − *C*) × 100/*C*_0_where ‘*C*_0_’ is the initial paracetamol concentration (mg L^−1^) and ‘*C*’ is the paracetamol concentration (mg L^−1^) in solution after degradation.

## 3 Results and discussion

### 3.1 Characterizations analysis

**Fig. 1:**
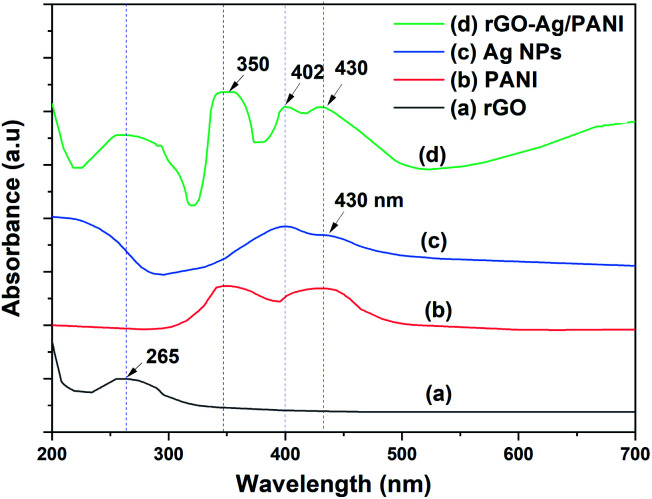
The UV-Visible absorption spectra of (a) rGO, (b) Ag NPs, (c) PANI, and (d) rGO–Ag/PANI nanocomposites.

The UV-Visible spectra of rGO, Ag NPs, PANI, and rGO–Ag/PANI are presented in Fig. 1. The characteristic peak of π → π (C=C) electronic transition of rGO is observed at 265 nm, which shows the partial restoration of the carbon framework by the reduction of GO.^24^ PANI shows peaks at 350 nm, 430 nm, and a broad peak at 800 nm, which are due to the protonation of PANI dispersion in the acidic medium.^25^ Ag NPs show two surface plasmon resonance (SPR) absorption peaks at 400 nm and 430 nm. The SPR absorption bands are varied depending on the size and shape of the Ag NPs.^26^ Hence, the SPR peaks of low particle size Ag NPs could be observed at 400 nm and the larger one at 430 nm. The spectrum of rGO–Ag/PANI showed peaks at 260 nm, 350 nm, 400 nm, and 430 nm. These peaks correspond to rGO (260 nm), PANI (350 nm and 430 nm), and Ag NPs (400 nm and 430 nm). Presence of all the peaks confirms the coexistence of all the composites without losing their electronic structure. The optical bandgap of rGO–Ag/PANI was measured by UV-Vis diffuse reflectance spectroscopy (UV-DRS). As shown in Fig. S1,† the bandgap was calculated to be 3.2 eV, which is equal to the bandgap of pure PANI. This shows that the PANI composite with Ag and rGO did not lower the bandgap.

**Fig. 2:**
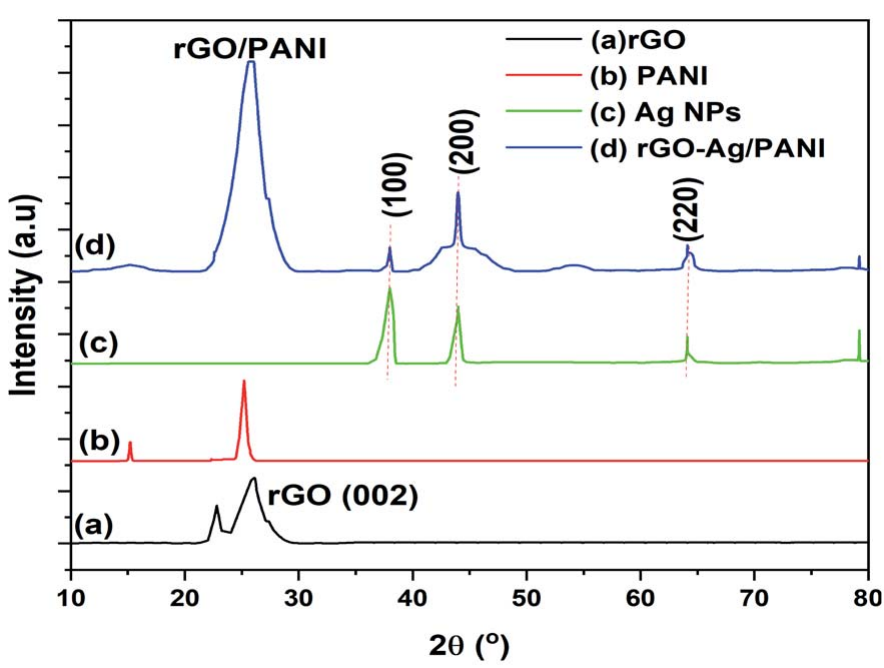
The XRD patterns of (a) rGO, (b) Ag NPS, (c) PANI, and (d) rGO–Ag/PANI nanocomposites.

Fig. 2 shows the powder XRD patterns of rGO, PANI, Ag NPs, and rGO–Ag/PANI nanocomposites. As is shown in Fig. 2(a), the broad peak at 2*θ* = 26.1° is attributed to the (002) and *d*-spacing of 3.4 Å, thus indicating the formation of rGO.^27^ The weak intense peak at 15.2° and strong intense peak at 25° indicates that the PANI is in crystalline structure (*d*-spacing 7.2 and 3.5 Å).^28^ The Ag NPs shows peaks centered at 2*θ* = 38°, 43.999°, 64.1°, and 79.2° corresponding to the (111), (200), (220), and (311) lattice indices (JCPDS file no. 04-0783), respectively.^29^ In the rGO–Ag/PANI nanocomposites, the intense broad peak observed at 2*θ* = 25° could be the merged peak of rGO and PANI. The peaks at 38°, 43.999°, 64.1°, and 79.2° correspond to the (111), (200), (220), and (311) of the Ag nanoparticles. Thus, XRD studies reveal the presence of rGO, PANI, and Ag NPs.

**Fig. 3:**
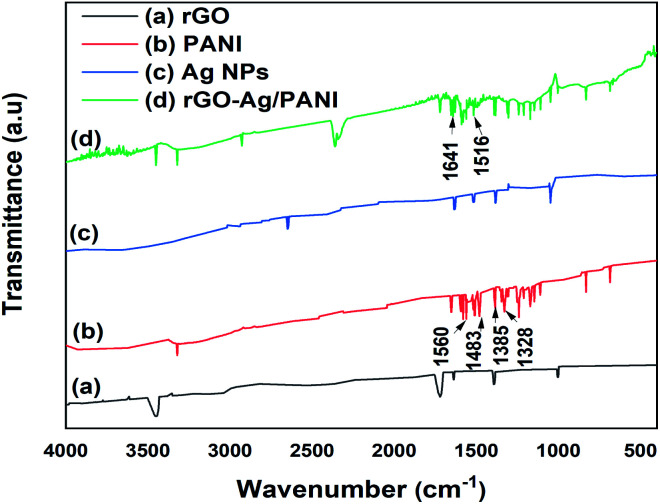
FTIR of (a) rGO, (b) Ag NPs, (c) PANI (d) rGO–Ag/PANI nanocomposites.

The structural information of the rGO–Ag/PANI nanocomposites is explored by FTIR (Fig. 3). Moreover, rGO absorption peaks at 1720 cm^−1^ (C=O stretching), 1640 cm^−1^ (C=C stretching), 1390 cm^−1^ and 3450 cm^−1^ (–OH deformation and stretching) and 1002 cm^−1^ (C–O stretching) were also observed.^30^ The PANI spectrum has ring-stretching vibrations (C=C) of the quinoid and benzenoid at 1560 and 1483 cm^−1^, respectively.^31^ Moreover, the C–N stretching vibration of quinoid and benzenoid rings are shown at 1385 cm^−1^ and 1328 cm^−1^, respectively.^32^ The N–H bending bands (1304 cm^−1^, 1210 cm^−1^ and between 1515–1520 cm^−1^), and nitro group of *o*-nitroaniline due to asymmetric and symmetric stretching (1510 and 1346 cm^−1^) are also detected.^33^ The rGO–Ag/PANI shows the peak due to PANI and rGO; however, the band positions and intensities are found to differ. The C=C stretching vibrations of the quinoid and benzenoid found at 1560 and 1483 cm^−1^ are shifted to 1641 cm^−1^ and 1516 cm^−1^, respectively. This could be explained by the electrons from rGO and Ag atoms conjugate to the quinoid and benzenoid rings, which contributed to the shift in the FTIR spectrum of rGO–Ag/PANI.^31^ Moreover, the C=O band of rGO shows reduced intensity in the composites than the pristine rGO. The decreased intensity of C=O ascribes the synergistic role of the composites for the reduction of C=O.

**Fig. 4:**
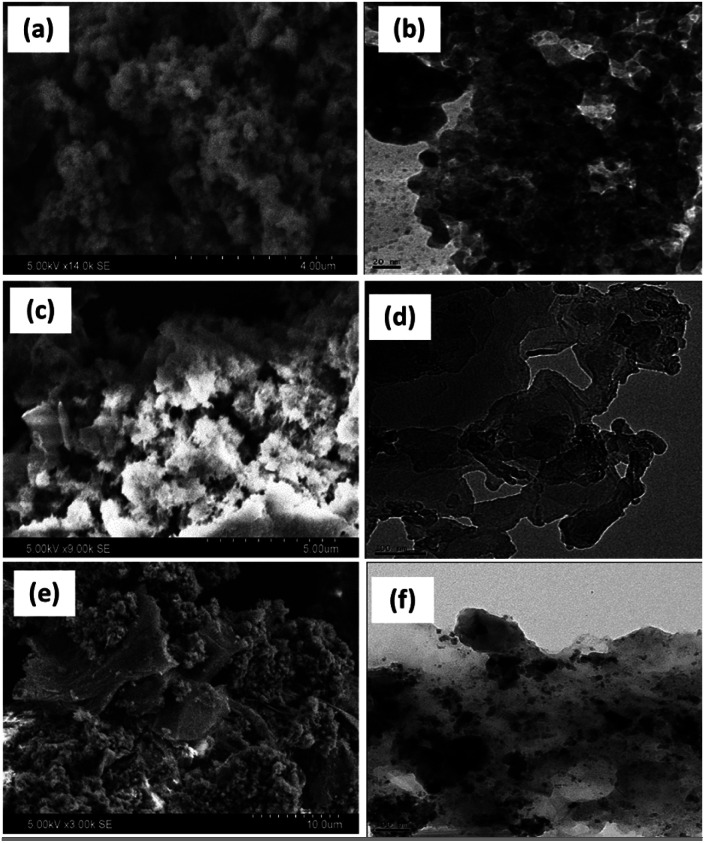
The representative SEM and TEM images ofPANI (a and b), PANI–Ag (c and d), and (e and f) rGO–Ag/PANI nanocomposites, respectively.

Fig. 4 shows the SEM and their corresponding TEM images of PANI, PANI–Ag, and rGO–Ag/PANI nanocomposites. The low magnification SEM micrograph shows high surface porosity of PANI. PANI brings flake-like morphology while it makes the composite with Ag NPs. Furthermore, the SEM image of rGO–Ag/PANI shows the cluster of PANI–Ag deposited on the rGO sheet. Similarly, the TEM images of rGO–Ag/PANI indicate the Ag NPs onto the PANI/rGO matrix. As shown in Fig. 4(f), the Ag NPs are finely distributed in the composite with an average particle size ranging from 2 nm to 10 nm. Besides, there are limited aggregates of Ag NPs, as shown in the composite.

The surface composition of rGO–Ag/PANI composite was further investigated by X-ray photoelectron spectroscopic (XPS) analysis. The survey spectrum shown in Fig. S2a† confirmed the existence of the elements Ag, C, S, N, and O. In Fig. S2b,† the presence of Ag in the rGO–Ag/PANI was exhibited in the XPS spectrum, in which the peaks located at 367.49 eV and 374 eV correspond to Ag 3d_3/2_ and Ag 3d_5/2_, respectively, in the Ag 3d spectrum. The C 1s dominant peak at 284.95 eV could be attributed to the binding energy of sp^2^ C=C bonds of rGO. Similarly, prominent peaks at 400.75 eV, 532.55 eV and 169.43 eV were observed due to N 1s, O 2s, and S 2p, respectively.

The adsorption–desorption isotherm plot and BET plot of PANI and rGO–Ag/PANI composites are shown in Fig. S3.† The mean pore diameter and specific surface area of PANI were found to be 20.272 nm and 1.6951 × 10^1^ (m^2^ g^−1^), respectively. The composite shows smaller pore diameter (6.6489 nm) and specific surface area (5.3866 × 10^0^ m^2^ g^−1^) compared to that of PANI. Fig. S3(a and c)† reveals that N_2_ adsorption and desorption isotherm presents a hysteresis of type IV, typical of mesoporous materials.

### 3.2 Photocatalytic degradation analysis

The photocatalytic activity of rGO–Ag/PANI nanocomposites were investigated *via* degradation of paracetamol under visible light irradiation. The degradation is carried out at various reaction parameters such as pH, the concentration of rGO–Ag/PANI, and presence of H_2_O_2_.

#### Effect of pH of the solution

**Fig. 5:**
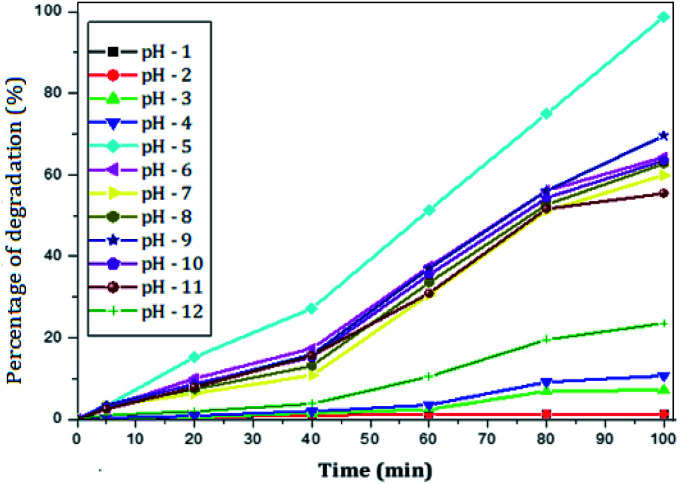
Effect of pH on the percentage of degradation of paracetamol (25 mg L^−1^) at a fixed amount of rGO–Ag/PANI (50 mg), and room temperature (300 K).

The effect of pH on the degradation of paracetamol (25 mg L^−1^) using rGO–Ag/PANI (50 mg) was studied at varying pH of the solution ranging from 1 to 12. Fig. 5 shows the effect of pH on the degradation of paracetamol. The maximum removal was obtained at pH 5 and for pH below 5 the degradation of paracetamol becomes less. The pH values of 6, 7, 8, 9, 10 and 11 were found to yield a mild response. Yet, extremely high pH (12) is not suitable for the degradation of paracetamol. The absorption spectrum in the acidic and the basic medium is presented in Fig. S4 and S5.†

#### Effect of rGO–Ag/PANI dose

**Fig. 6:**
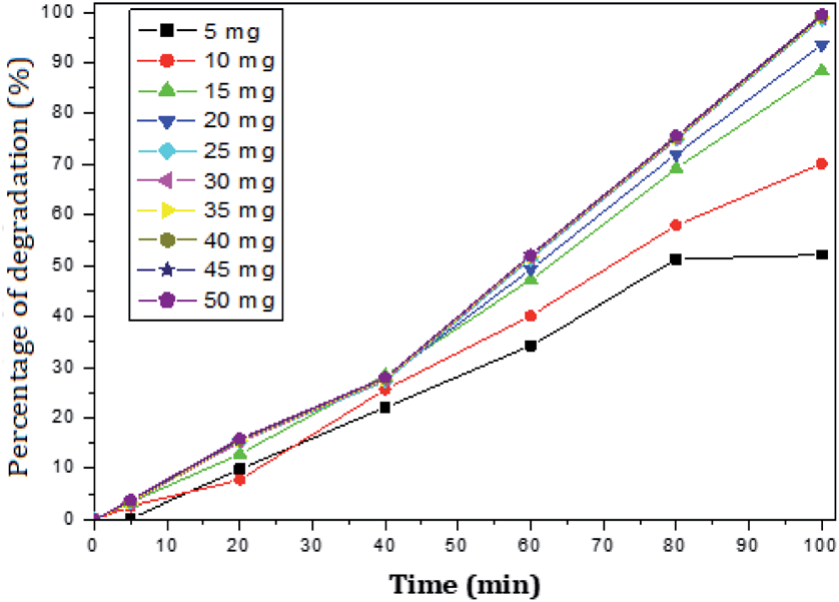
Percentage of degradation of paracetamol (25 mg L^−1^) with variation in concentration of nanocomposite in acidic medium (pH 5).

**Fig. 7:**
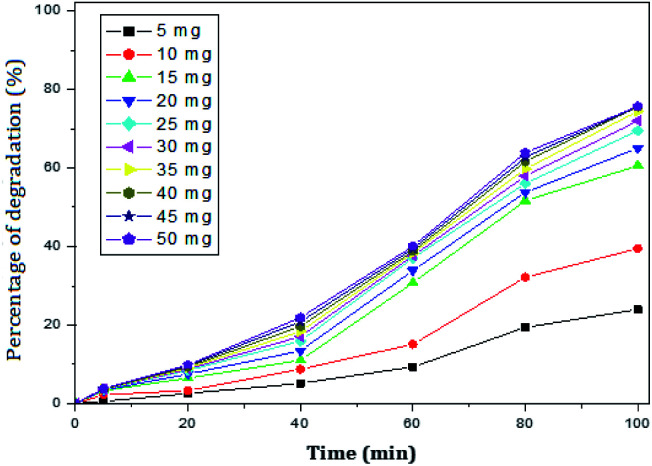
Percentage of degradation of paracetamol (25 mg L^−1^) with variation in concentration of nanocomposite in basic medium (pH 9).

The degradation of paracetamol was studied with respect to the change in concentration of the nanocomposites at pH – 5 and 9. The concentration of paracetamol was constant (25 mg L^−1^) and the weight of nanocomposites was varied from 5 mg to 50 mg. The maximum percent degradation of paracetamol was achieved at rGO–Ag/PANI dose ranging from 25 mg to 50 mg (Fig. 6). As shown in Fig. 6, all the degradation (%) points from 25 mg to 50 mg coincide onto each other. This confirms that an excessive dose of the composites may not bring additional degradation efficiency. Moreover, 15 mg and 20 mg dose of the photocatalyst cannot adequately degrade paracetamol. Therefore, 25 mg of rGO–Ag/PANI could be reasonably recommended for the degradation of 50 mL paracetamol (25 mg L^−1^) at pH 5. The removal of paracetamol was investigated at pH 9 with varying doses of rGO–Ag/PANI (Fig. 7). Similar trends were obtained as in the acid solution, but the overall performance of the composites was found to be poor at this pH.

#### Effect of paracetamol concentration

**Fig. 8:**
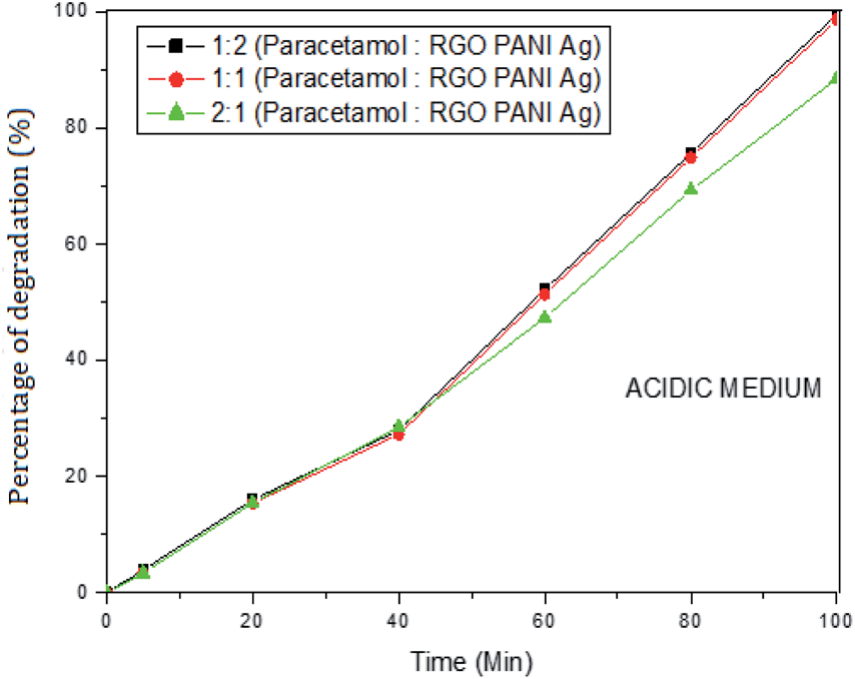
The effect of paracetamol dose on the removal efficiency of rGO–Ag/PANI at pH 5.

**Fig. 9:**
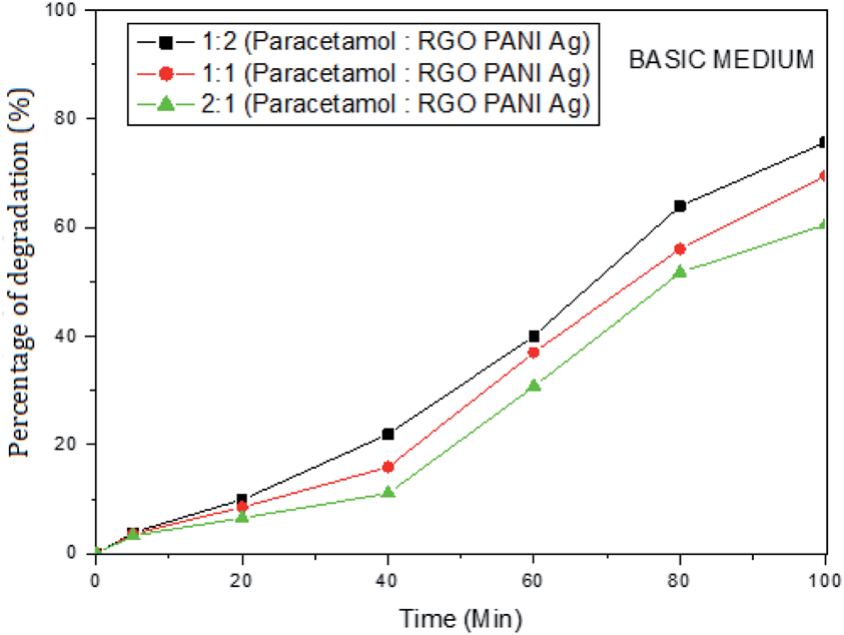
The effect of paracetamol dose on the removal efficiency of rGO–Ag/PANI at pH 9.

The varying ratio of paracetamol to rGO–Ag/PANI dose was used to investigate the effect of paracetamol concentration on the degradation efficiency. The effect was studied both in the acidic (pH 5) and basic (pH 9) mediums. Fig. 8 shows the effect of paracetamol dose at pH 5. The results show that the efficiency of rGO–Ag/PANI increases with the decrease of paracetamol concentration. Maximum removal was found when 25 mg L^−1^ of paracetamol reacted with 25 mg of rGO–Ag/PANI in a 50 mL solution. Similar results were obtained at pH 9 (Fig. 9).

### 3.3 Mechanism of paracetamol degradation using rGO/Ag/PANI

**Fig. 10:**
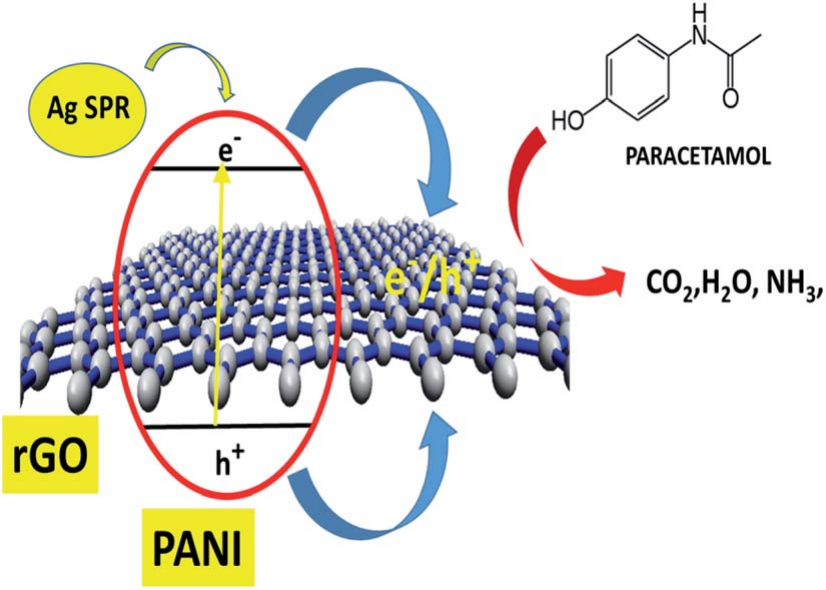
The plausible degradation mechanism of paracetamol using rGO–Ag/PANI.

The overall synergistic degradation of paracetamol by rGO, PANI, and Ag can be plausibly explained, as shown in Fig. 10. The rGO mainly acts as a binder and supports the charge separation. Because of the sp^2^ hybridization on each C-center, there is extended delocalization of electron transfer that helps for a sink of photoejected electrons. The π to π overlapping of paracetamol to the rGO sheet could enhance the adsorption, which is an important step in the photocatalysis process. PANI is known as a p-type semiconductor and absorbs light in the visible region.^15^ Therefore, PANI forms active charge carriers [hole (h^+^) and electrons (e^−^)] during illumination with visible light. Consequently, h^+^ and e^−^ participate in the degradation of paracetamol. Ag NPs play a significant role as a catalyst for the degradation of paracetamol. More importantly, the SPR absorption of light in the visible region creates an electron gradient to the VB of PANI. Therefore, the overall synergy of the rGO–Ag/PANI is explained by strong adsorption efficiency, charge separation, and light absorption in the visible region.

### 3.4 Recyclability of rGO–Ag/PANI

**Fig. 11:**
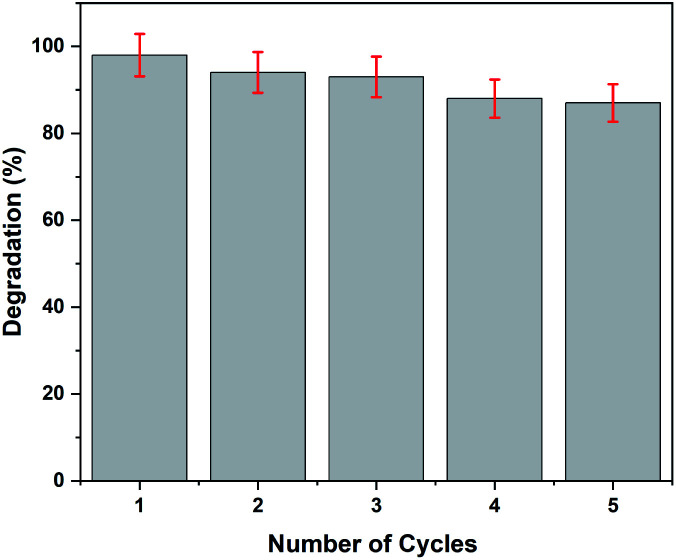
The recyclability test of rGO–Ag/PANI for the degradation of paracetamol.

The recoverability test is crucial for the practical application of the photocatalyst. Herein, five successive cycles of photocatalytic degradation of paracetamol were carried out to evaluate the recyclability of rGO–Ag/PANI. The optimum degradation condition (50 mg rGO–Ag/PANI, 25 mg L^−1^ paracetamol at pH 5, and room temperature) was used for the recyclability test. After each consecutive cycle, the photocatalyst was collected, washed at least three times, and dried in the oven for further use. As is shown in Fig. 11, within a 5% error, the rGO–Ag/PANI nanocomposites were successfully reused for five successive adsorption cycles. This indicates the potential of rGO–Ag/PANI to be reused for successive experiments.

## 4 Conclusions

A facile synthesis of rGO–Ag/PANI nanocomposites was achieved using vitamin C as a reducing agent. The UV-Visible, XRD, and FTIR analysis shows the formation of rGO–Ag/PANI nanocomposites. Supporting of the rGO layer to the cluster of PANI–Ag was observed by SEM and TEM analysis. The synergistic photocatalytic efficiency of the nanocomposites was found to be promising for the degradation of paracetamol. The results showed that 99.6% paracetamol was degraded at the optimum condition, *i.e.*, pH 5, 25 mg rGO–Ag/PANI, 50 mL of 25 mg L^−1^ paracetamol, and room temperature (300 K). The remarkable degradation efficiency of the composites is obtained due to the synergistic effect of rGO as charge separation, PANI as the formation of e^−^/h^+^ pair in the visible region, and Ag as the production of e^−^s and active catalyst surface.

## Conflicts of interest

There are no conflicts to declare.

## Supplementary Material

RA-011-D1RA00171J-s001
