# Supplementary material for: Vitamin C assisted synthesis of rGO–Ag/PANI nanocomposites for improved photocatalytic degradation of pharmaceutical wastes
Source: RSC Adv. 2021 Mar 24;11(20):12030–5. doi: 10.1039/d1ra00171j (PMC8697029; doi:10.1039/d1ra00171j)
Supplement: RA-011-D1RA00171J-s001 [file RA-011-D1RA00171J-s001.pdf]

Electronic Supplementary Material (ESI) for RSC Advances.
This journal is © The Royal Society of Chemistry 2021

SI

Vitamin C assisted Synthesis of rGO-Ag /PANI Nanocomposites for Improved Photocatalytic Degradation of Pharmaceutical Wastes

S-1: synthesis of grapheme oxide (GO)

The GO was synthesized from Graphite by Modified Hummers' process. In typical procedure, accurately 6 grams of graphite flakes were dissolved in a round bottom flask contains 50 mL of acetone under sonication for 30 minutes. The obtained graphite residue was dried at 50 ^{0}C for two hours. Then dried and sonicated graphite (6 g) and $NaNO_3$ (3.0 g) were suspended in 139 mL concentrated sulphuric acid in an ice bath (below 5 ^{0}C) in a 500 mL round bottom flask under gentle magnetic stirring for 20 minutes. Then, 18 g of $KMnO_4$ was slowly and continuously added at 20 ^{0}C. Then, the ice bath was removed and the solution is continuously stirred and refluxed for 3 hours at 35 ^{0}C. After that, 250 mL of distilled water was added under constant stirring for1 hour. The excess unreacted $KMnO_4$ was removed by 25 mL of 30 % hydrogen peroxide. The complete removal of $KMnO_4$ was indicated by a colour change of solution to yellow. The prepared GO was carefully washed with deionized water 3 to 4 times and dried overnight in the oven at 50 ^{0}C.

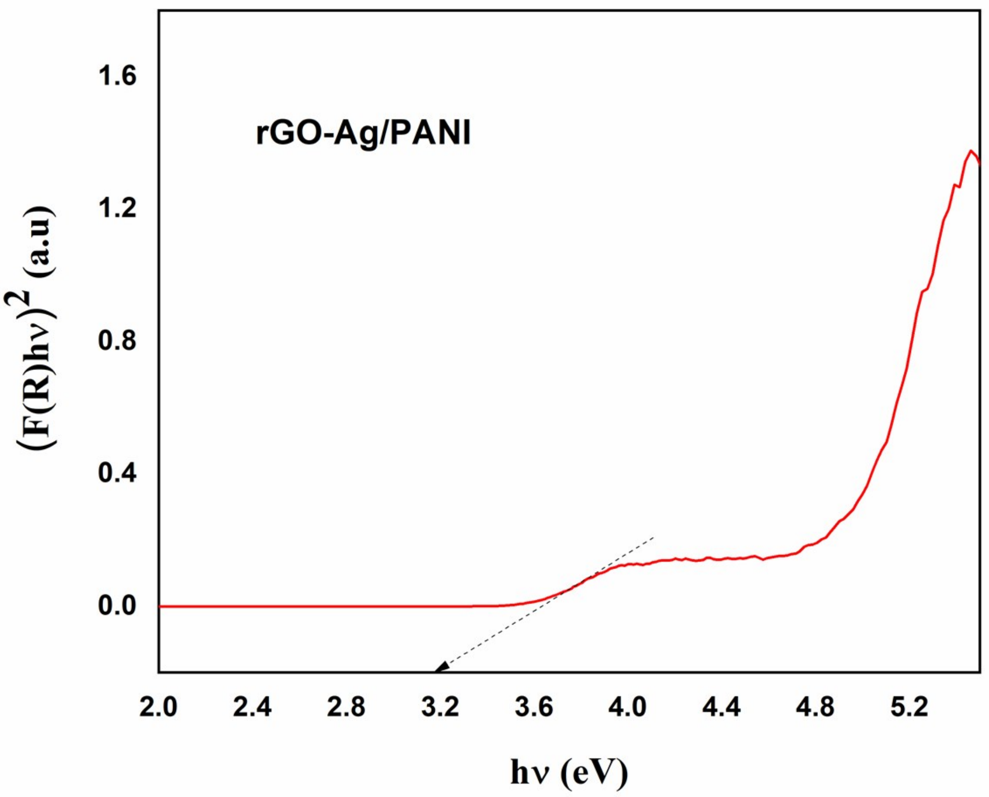

SI Figure 1: The UV RDS spectrum of rGO-Ag/PANI nanocomposite.

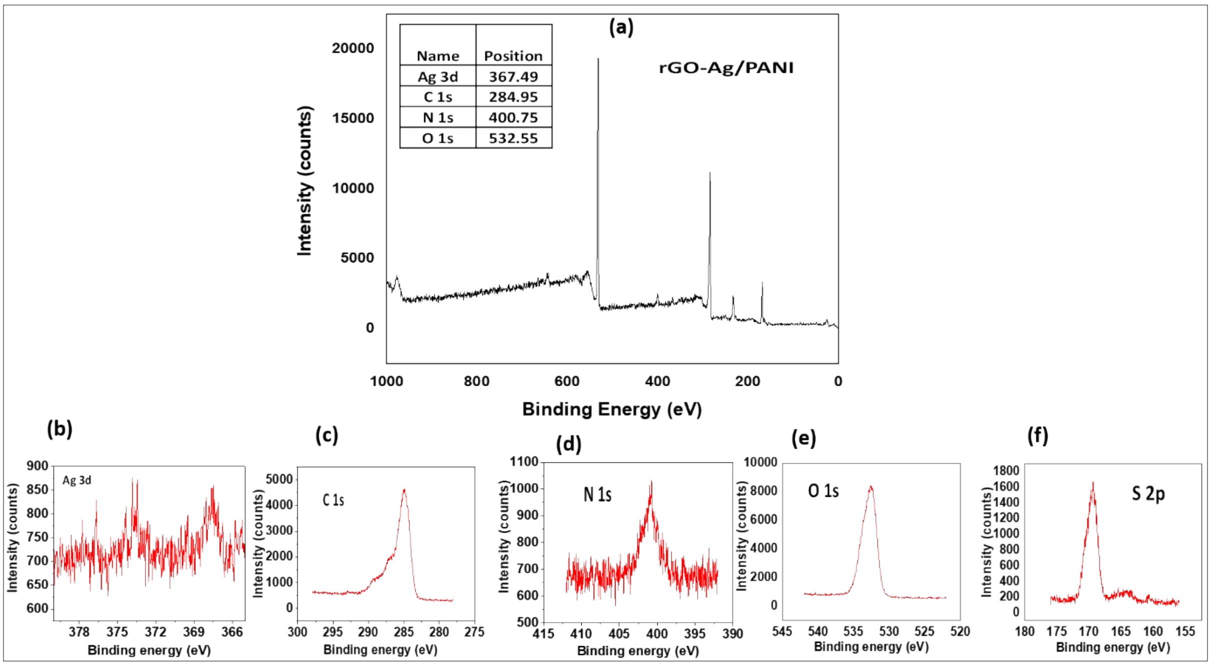

SI Figure 2: The XPS analysis of rGO-Ag/PANI nanocomposites (a) the survey spectrum and (b-f) high-resolution elemental XPS spectrum.

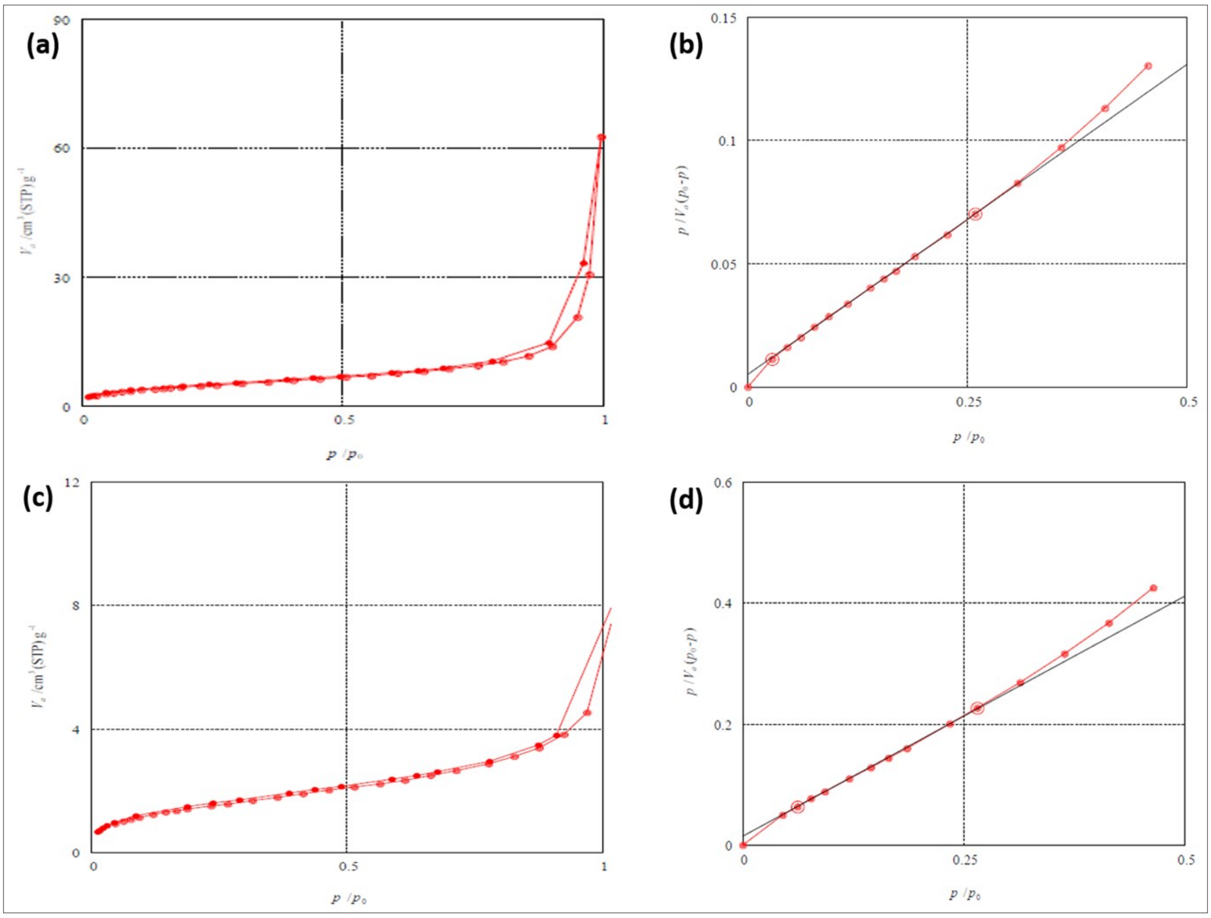

SI Figure 3: The Adsorption/desorption isotherm plot (a and c) and BET plot (b and d) of PANI and rGO-Ag/PANI composites, respectively.

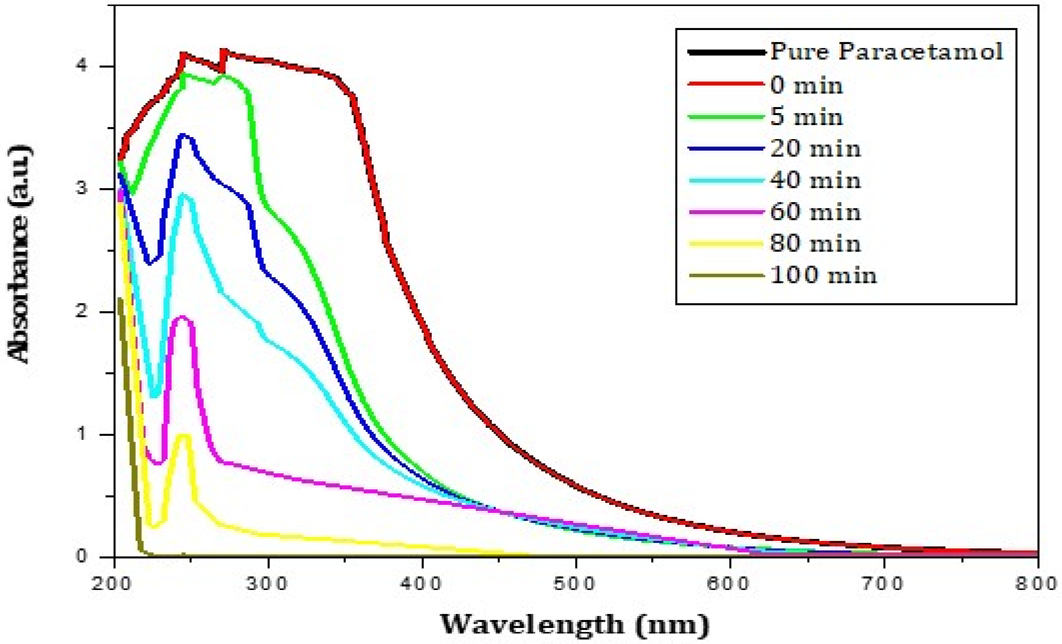

SI Figure 4: Percentage of degradation of Paracetamol (25 mg) and nanocomposite (50 mg) in acidic medium

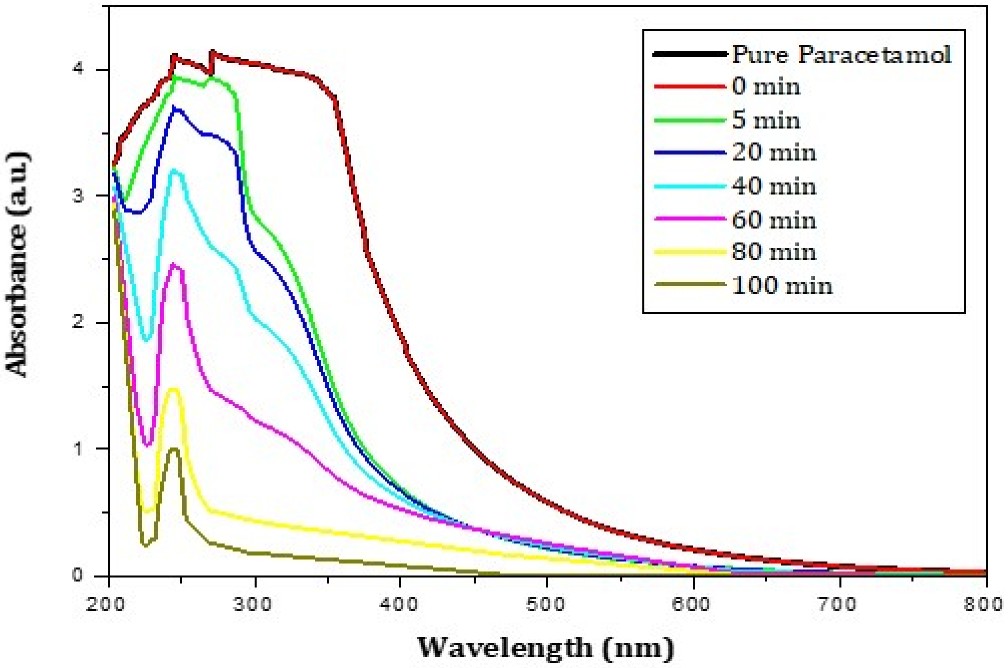

SI Figure 5: Percentage of degradation of Paracetamol (25 mg) and nanocomposite (50 mg) in basic medium
